# Meaning of the Solid and Liquid Fascia to Reconsider the Model of Biotensegrity

**DOI:** 10.7759/cureus.2922

**Published:** 2018-07-05

**Authors:** Bruno Bordoni, David Lintonbon, Bruno Morabito

**Affiliations:** 1 Cardiology, Foundation Don Carlo Gnocchi Irccs/department of Cardiology, Institute of Hospitalization and Care, Milano, ITA; 2 Osteopathic Technique, London School of Osteopathy, London, GBR; 3 Osteopathy, School of Osteopathic Centre for Research and Studies, Rome, ITA

**Keywords:** fascia, myofascial, biotensegrity, manual therapy, osteopathic, chiropractic

## Abstract

The definition of fascia includes tissues of mesodermal derivation considered as specialized connective tissues: the blood and lymph. As water shapes rocks, bodily fluids modify the shape and functioning of bodily structures. Bodily fluids are silent witnesses to mechanotransductive information, allowing adaptation and life, transporting biochemical and hormonal signals. While the solid fascial tissue divides, supports, and connects the different parts of the body system, the liquid fascial tissue feeds and transports messages for the solid fascia. This article reconsiders the model of biotensegrity, by revising the definition of solid and liquid fascia, and tries to integrate the fascial continuum with the lymph and blood in a new model, because in the previous model, these two liquid elements were not taken into consideration. The name given to this new model is Rapid Adaptability of Internal Network (RAIN).

## Introduction and background

From an embryological perspective, the fascia originates in the mesoderm, although, according to some authors, this connective network can be partially found in neural crests (ectoderm), with particular reference to the cranial and cervical areas. All the tissues considered as “specialised connective tissues” of mesodermal derivation, such as blood, bone, cartilage, adipose tissue, hematopoietic tissue, and lymphatic tissue, are regarded as part of the fascial system [1]. The ordinary movements of the body are possible thanks to the presence of fascial tissues and their inseparable interconnection that allows the sliding of the muscular framework, the sliding of nerves and vessels between contractile districts and joints, as well as all the sliding and movements of organs, as influenced by the position of the body [1]. The fascial continuum allows for the correct distribution of the tensional information produced by different tissues enveloped and supported by the fascia, so that the whole body system can interact in real time [1]. The fundamental characteristics of the fascia is the ability to adapt to mechanic stress, remodel the cellular/tissue structure, and mirror the functional necessity of the environment where the tissue is located [2]. Of the scientific data deriving from cellular microscopy, we can avoid citing the anatomic subdivision of the different fascial depths (superficial and deep), because we know that a clear separation of the structures does not exist in vivo; instead, there is a functional continuum [2]. We can subdivide the fascial tissue into solid and liquid fascia. The first includes all that is considered connective tissue, while the second only includes what is considered specialized connective tissue: blood and lymph [3].

## Review

The discussion will examine existing knowledge on solid and liquid fascia and the concept of biotensegrity of solid and liquid fascia. Finally, we will formulate a hypothesis that integrates solid and liquid fascia in the concept of biotensegrity.

Solid fascia

Collagen makes up more than the 30% of the protein mass in the human body. Its most common shape is the collagen fibril, made up of around 300-nm of tropocollagen (polypeptide triple helices). The fibril is highly organized and provides the framework for the extracellular matrix (ECM), tendons, bones, and other load-bearing structures [4]. Collagen fibrils resemble self-assembling cables on a nanometric scale. The biosynthesis of the collagen takes place thanks to different types of cells, depending on the kind of tissue. For example, osteoblasts form collagen in bones, while fibroblasts form collagen in tendons [2]. There are different types of connective tissue, classified according to some morphologic and functional criteria. We find dense connective tissues (fibrous or elastic), where collagen is arranged in regular and irregular structures, and loose connective tissues (fibrous, reticular, or elastic), which stands out because of the abundance of amorphous substances compared to the quantity of fibrous components [5]. In dense connective tissue, we mainly find collagen types I, III, XII and XIV, and elastin, while in loose connective tissue, we find collagen types I, III, IV, V, VII, XII, and XIV [5]. Fibroblasts are the main cellular component of  connective tissues and secrete components of ECM such as collagen and matrix, glycosaminoglycan (GAG), elastic and reticular fibres, and glycoproteins. Fibroblasts communicate with each other and are fundamental for managing perceived and produced tension [6]. They play a fundamental role in conveying tension, and can dynamically affect mechanical tension, rapidly remodeling their cytoskeletons; the fibroblast’s cytoskeleton is made of microtubules, namely actin filaments and intermediate filaments; specifically, the flexibility of actin allows fibroblasts to adapt more rapidly in the presence of compressive forces, due to the lengthening of the fascia [6]. If the mechanical information is present for only a short period of time, any morphological variation is reversible, and the cytoskeleton of the fibroblast can be restored to its original state. The fibroblasts play a significant and active role in stimulating inflammatory processes, because they are responsible for cleaning, repairing, and replacing the elements of the fascial continuum that have been and are affected by traumas resulting from daily use [6]. Fibroblasts largely constitute the microvacueoles. Microvacueoles have a diameter ranging from a few microns up to 200 nanometers, with their size probably varying depending on the cells they constitute and/or the body area. The fibrils vary in size between 5 and 70 nanometers in diameter, reaching a length of 10-100 nanometers [2]. About 70% of these fibrils are constituted by collagen type I, III and IV, and about 20% by elastin, with around 4% by lipids. The microvacueoles are rich in water, thanks to the hydrophilic properties of the lipids and, in particular, of proteoglycans (approximately 72%). The core of these molecules is a protein with one or more covalent bonds with polysaccharides (glycosaminoglycans - GAGs); the negative charge of GAGs attracts water molecules, facilitating their passage through the membrane of the microvacueoles, and ensuring hydration. Hydration helps in maintaining the pressure and volume [2]. Connective tissues derive from the mesenchyme [7]. During embryonal development, connective tissues probably influence the shape (morphogenesis) of the structures that they will contain and connect. Embryonal mesenchyme or embryonal connective or undifferentiated mesenchyme tissues are made up of branched star-shaped cells with a high mitotic rate (high reproductive ability); they are considered pluripotent stem cells, because they have the ability to differentiate into different tissues. The embryonal mesenchyme is the source of not only many connectival structures, but also of stromal stem cells; over the course of the development process, they occupy the spaces between germ layers, connecting various structures, and constituting the stroma of organs [8]. Mesenchyme is present in and derives from all of the three embryological layers (ectoderm, mesoderm, and endoderm), especially from the mesoderm and ectoderm [9-10]. The fascia that forms part of the head (muscles, bones, skin, and so on) and part of the cervical spine derive from the mesoderm and ectoderm [2]. Fascial tissues are described as layered, but it is a widespread habit that comes from anatomic dissection. The layers are inseparable and they move and answer/react in unison to the presence of mechanical/metabolic information [2].

Liquid fascia

Blood and lymph derive from the mesoderm and are considered to be connectival tissues [3]. Blood and lymphatic vessels are solid fascial structures; what they carry is liquid fascia. In addition to its nutritive functions, blood also provides a way of linking different organs which can communicate with each other with hormones and chemical mediators, guaranteeing the integration of the functions of the organism. It is the vehicle of immune cells and platelets, and it can reach places where their presence is necessary (for example, areas of inflammation), of antibodies and proteins of the clotting system, and of the numerous transport proteins (such as lipoproteins, transferrin, ceruloplasmin and albumin) to which the water-insoluble compounds that circulate in blood are attached [11]. Human blood is a liquid that can be ruby red (clean), or purplish red (dirty); its viscosity is around four times higher than the viscosity of water, its specific weight is 1,041-1,062 g/cm^3^. It makes up around 7.7% of the human body weight, its temperature is around 37/38 °C (it varies depending on internal and external factors), and its pH (in arteries) is 7.38-7.42 (the pH of an optimal saline solution should be 7.383). In men, it is made up of a liquid part (55%) called plasma, and a corpuscular part (45%) that consists of cells or cell fragments (average values for a healthy adult male);  in women, the liquid part takes up 60% and the corpuscular part takes up 40%. This ratio is called hematocrit and evaluates the volume of corpuscular blood elements under normal conditions [11]. Plasma is a pale-yellow liquid made up of water (90%), organic substances, and dissolved salts (10%). Blood is a connective tissue [5]. It consists of cells and cell fragments in suspension in an extracellular matrix of complex composition. The unusual characteristic of blood is the fact that the extracellular matrix is a liquid, which means that blood is a fluid connective tissue. In blood, there are two different components that can be separated by centrifugation: a fluid matrix called plasma, and corpuscles, which are cells or cell fragments [11]. Corpuscles are of three kinds: erythrocytes, platelets, and leukocytes. Only leukocytes are complete cells; erythrocytes are anucleate cells and platelets are cell fragments. Erythrocytes are present in larger quantities than the other elements, which is why they influence the value of the hematocrit much more than leukocytes or platelets, which make up around 1% of the total volume. Erythrocytes, just like the other elements, are generated by pluripotent stem cells located in the bone marrow, particularly in the ribs, sternum, pelvis, and vertebrae. There are different kinds of leukocytes. Granulocytes are characterized by the presence of big granules in the cytoplasm. They are visible under an optical microscope, and after colouring can be divided into neutrophils (with an affinity to neutral colouring), eosinophils (affinity to acid colouring), and basophils (affinity to basic colouring) [12]. Lymphocytes, which include lymphocytes T, lymphocytes B, and natural killer cells, participate in specific defence roles: they recognize a pathogen, target it, and then attack it. This targeting implies almost always the production of proteins circulating in the blood, called antibodies. Monocytes are the biggest leukocytes, characterized by a big horseshoe-shaped nucleus [12].

The lymphatic system effectively removes the excess of interstitial fluids, solutes, and various cells, guiding them towards the bloodstream, maintaining the volume of plasma and interstitial fluids in constant balance [11]. The lymphatic system originates from the interstitial tissue called “initial lymphatics”, small capillaries delimited by discontinuous endothelium and basement membrane, offering resistance to the flow of fluids and substances (hydrophiles molecules, cells, viruses, and bacteria). They attach to the external surface of cells through collagen fibrils (collagen type VII) [11]. This collagen allows the transmission of mechanical forces towards the lumen of the lymphatic vessel; there are autonomous contractions in some vessels, thanks to the presence of filaments similar to actin. These initial lymphatics become wider, creating collecting ducts that consist of collagen and smooth muscle cells and elastic fibres [11]. Lymphatic vessels have got their own tone and, probably, their own intrinsic contraction autonomy; according to recent data, they also exhibit sensibility to flow variation (sensory functions). They are surrounded by nerves of the autonomous system (mainly sympathetic fibres), which could help better coordinate lymphatic transport. Lymphatic vessels adapt and change their elastic capacity, improving or worsening the function of lymphatic transport [13]. Primary lymph valves are formed by the cytoplasmic extent of the adjacent endothelial cells linked by close connections. The valves of these cells protrude towards the inside; this way, what goes in can’t go out. Finally, there are interluminal valves (weaker): two sheets attached to the opposite sides of the lymphatic vessel and connected to zonules (perimeter junction involving a band that surrounds the cell) [11]. Lymph flows thanks to external mechanical compressions (e.g., muscle contractions) and its own intrinsic contraction abilities. The lymphatic system is subject to aging, losing its elasticity and creating “aneurysms” over time, or the number of blood vessels or  lymphangions (the lymphatic functional unit) decrease. Recent evidence reveals that lymphatic vessels are supported by a nervous system, of vagal cholinergic type and sympathetic type, able to modulate the contraction (peristalsis, also helped by the breathing and pulsation of arteries) of vessels endowed with contractile fibres (with an actin-like protein) [11]. These thin nerves reach the external layer of the lymphatic vessel and then reach the deepest endothelial layer; this nerve network deteriorates in elderly people. The parasympathetic and sympathetic systems acts not only as tension or vessel tone modulators, but also as sensors of the contractile layer of the vessel itself [13]. The dural system has a lymphatic system called the glymphatic system. The cerebrospinal fluid (CSF) is not only drained through the venous system, but also through the lymphatic system [14]. The lymphatic dural vessels emerge from the skull following the reverse path of the pterygopalatine artery and a branch of the internal carotid artery, following the venous pathways and the cranial nerves exiting the skull [14]. Lymphatic vessels follow the vein paths of the cribriform plate towards the nasal mucosa, following the ways the cerebrospinal fluid (CSF) exits [14]. The glymphatic system absorbs the interstitial liquid and the cerebrospinal fluid (CSF) from the subarachnoid space and transports it outside of the skull, more specifically from the base, up to the cervical spine. This mechanism is stronger during sleep [14].

Biotensegrity

Tensegrity, the tension that preserves integrity, comes from a principle of architecture and was proposed by Fuller in 1961 [15]. A structure is stabilized by the balance of constant tension and by the presence of a discontinuous compression. This organization is self-stabilizing, allowing it to manage tension variations with a certain degree of flexibility, transferring the forces applied to the whole structure [15]. The architectonic principle can be found in the living, from the body system to the single cell. The first transposition of the concept to the body system dates back to 1977, when the tensegretive vision was applied to the column [15]. In 2007, the vision of the column as a tensegretive structure was taken into consideration again, and the biotensegretive model was suggested. The model suggests the presence of the principle of self-regulation in the living, where constant tension is represented by musculature, and bones/joints play the role of discontinuous compression [16]. The concept of biotensegrity as applied to the cell was first proposed by Ingber in 1993 [17]. The biotensegretive model allows the cell or the system to sense external or internal forces, to transmit that mechanical information to the inside/outside of the cell or system, in order to allow local and systemic functions to remain stable. When a cell deforms at the passing of a force vector, the change of shape activates a series of metabolic and hormonal events that make the cell less fragile, allowing it to continue to function. This mechanism is called mechanotransduction [16]. We can summarize the principle of biotensegrity with a thought by Bernstein: “…the ability to find an action solution for any environmental situation – to solve adequately any emerging action problem.” [18]. The propagation of the mechanical information towards the cytoplasm and the deoxyribonucleic acid (DNA), deforming the cell at the passing of one or more force vectors, is an event as rapid as the speed of sound [18]. In the inside of the cell, we can find microfilaments, straight and tight as a rope, that form a geodesic triangular structure, and slightly bent microtubules. The microfilaments are the elements that provide constant tension, while the microtubules provide compression [15]. The ECM plays a key role in the transmission of force to and from the cell and in maintaining discontinuous compression in cooperation with the microtubules [15]. The ECM creates a three-dimensional net that surrounds all the cells, just like all the organs and tissues of the body [19]. It is made up of a ground substance or gel (glycosaminoglycans, hyaluronan, glycoproteins, and proteoglycans) and a fibrous framework (reticulum, elastin, and collagen fibres) in a way that helps maintain the tension of the cells of different tissues: ECM has its own biotensegrity. The fibrous framework acts as tensional element, while the base substance acts as discontinuous compression [20]. ECM communicates with the cell and its DNA via transmembrane proteins (integrin) gathered together to form focal adhesion complexes; this system allows the creation of an informational network for all tissues [20].

Biotensegrity of the solid fascia

The development of the biotensegretive model concerning the fascia comes from the observation of its influence on muscular coordination [18]. The fascial continuum envelopes and permeates muscles (epimysium, perimysium, and endomysium), it links them to one another, directly or indirectly through bones and joints, tendons and ligaments, allowing systemic communication and the transmission of force that involves the whole contractile tissue (with the cooperation of the neural system). There is transmission of intramuscular, intermuscular, and extramuscular force [21]. The myofascial tissue presents constant tension, while the joints and bones play the role of discontinuous compression [15, 18]. This organization allows for the real-time adaptation of posture and improvements in the expressed gesture: the fascial continuum acts in unison. The biotensegrity of the solid fascia allows it to constantly tune different body areas, according to the current necessity—this is a property the tension elements have, called “prestress” or “pretension” [15]. To summarize the concept of biotensegrity of the solid fascia, we quote Ingberg: “This is a physically integrated framework that supports the weight of our bodies, allows us to rapidly adjust to resist external forces, and permits us to move freely in our environment. But without the aid of surrounding tension-generating muscles and tension-resisting tendons, ligaments and fascia, bones and cartilage would do little to support our upright forms” [22].

Biotensegrity of the liquid fascia

Erythrocytes can change their shape according to the mechanical information they receive, and they can go back to their resting state at the cessation of the stress. Its membrane is made up of a double layer of lipids, reinforced by transmembrane proteins (spectrin family). These proteins connect the membrane with the cytoplasm, with a short actin filament, creating a complex net that reaches the DNA. The erythrocyte deforms according to the calibre of the vessel or according to the speed and direction of blood flow; the membrane changes its shape just like the cytoskeleton and, probably, thanks to the elastic accumulation of spectrins, the erythrocyte is restored to its original shape [23-24]. Its biotensegretive organization allows it to absorb the mechanical force it endures, to distribute it inside the cell, and to restore its shape, carrying out its function [15]. Actin and spectrins provide constant tension, while the double layer of lipids provides compression [25]. The ability to deform its shape is vital for its functioning, like distributing oxygen to tissues and retaining its viscosity even in narrow vessels. The capacity to correctly sense the present tension and change shape is probably due to the presence of some transmembrane proteins that act as ion channels (Piezo1, also known as Fam38a) [26]. The cell that represents the lymph the most is the lymphocyte, which changes its shape because it endures mechanical forces (flow speed, contractile tissues, etc.) and it follows the principle of mechanotransduction [27]. The cytoskeleton of the lymphocyte is very elastic, thanks to the presence of filaments like actin, alpha-actinin, calmodulins, spectrins, and myosin light chains [27]. The mechanical vector that deforms the cell reaches the nucleus, deforming it as well. The nucleus acts as a brake to prevent further deformation [27]. The shape of the lymphocytes is spherical, but with external stress, it can even change into a semicircle; with structures in prestress (cytoskeleton), mechanical hysteresis restores its original shape [27]. The membranes of the lymphocyte and of the nucleus represent the compression elements, while the filaments of the cytoskeleton are the factors that determine constant tension. The transmembrane proteins of the lymphocyte, like integrins, syndecans, and different ion channels (Ca2+activated potassium channels; voltage-gated Kv1.3; Ca(+) release-activated Ca(2+) or CRAC) make the process of mechanotransduction and the transmission of mechanical information to the inside of the lymphocyte easier [28-30]. The liquids of the body, like blood and lymph, cooperate for the well-being of the body’s health [31-32]. The lymph originates in the venous system, and it dies in the venous system, just like the venous system inevitably transforms into the arterial system. This is the continuum of the liquid fascia.

Rapid Adaptability of Internal Network (RAIN)

The human body is about 60% liquid; 40% of this liquid content is stored inside cells; the remaining 20% can be found in the interstices between cells. Just a small portion of the fluids is transported by vessels, like blood and lymph [31]. The arterial system transports biochemical and hormonal substances, including oxygen, to different tissues, with the ultimate aim of obtaining the optimal body synergy. Arterial capillaries are permeable and the plasma exits the vessel towards the cellular interstice of tissues; this is possible thanks to their hydrostatic pressure [31]. The venous system gathers waste products and water from tissues; the venous capillaries gather the biggest part of what is extravased from arterial capillaries, thanks to their oncotic pressure [31]. A small amount of plasma stays in the cellular interstice, and is collected, eventually, by lymphatic vessels, thanks to their low hydrostatic pressure; this final drainage is also useful to manage an excess accumulation of sodium, contrasting an imbalance of tension among liquids (hypertension) [31]. Blood and lymph determine the health of tissues and, consequently, their shape. This also happens during an ontological development. According to the embryologist Blechschmidt, during embryologic development, the pressure exerted by liquids (volume and speed), which each have different vectors and forces (direction), create the print and the direction for future tissues and organs [33]. During embryogenesis and in the fully-developed human body, the mechanical forces produced by the flows of blood and lymph or shear stress (laminar, turbulent, and oscillatory flows) also assure the preservation of the shape and function of the vessels (and of the heart) [31-34]. The pressure generated by the liquid fascia has three components: volume, direction, and speed. These are the components that determine the destiny of the shape and function of the solid fascia. If the solid fascia allows movement, the liquid fascia is the “intelligence” that allows its evolution and continuation.  The continuum of the liquid fascia is mirrored by the continuum of the solid fascia that forms the transport system: vessels. To give some examples, if a lymph node is negatively affected by pathology and therapy, like in the case of cancers and radartherapy, the whole lymphatic system is involved, implying the worsening of the transport of lymphatic material [35]. With aging, the whole vascular tree suffers from the thickening of vessels with an increasing in stiffness (in particular with the thickening of the intimal and adventitious layer), even in the absence of a manifest pathology like atherosclerosis: arteriosclerosis [36]. The venous system suffers a delay in its flow with aging, both in limbs and in the cerebral vascular system, with a high probability of developing different central and peripheral pathologies [37-38]. The tensegretive ability not only disappears, but when a local tensegretive discontinuity develops, the event will involve the whole blood and lymph net over time. If the solid fascia (vessels) is functional, the liquid fascia will be as well; if the liquid fascia does not encounter any obstacles in its path, the solid fascia (vessels and body structures) will be able to carry out its functions. This functional and biotensegretive synergy can be found in other examples, where the liquid fascia, as seen in the new theoretic model, acts as “joints” for the solid fascia. A joint allows the expression of muscular strength, thanks to tendons and ligaments that improve the lever arm and create a fundamental focal point for the biotensegretive musculoskeletal model. At the same time, constant tension elements (muscles) determine joint stability [39-41]. Compression and tension elements are vital to each other. Blood and lymphatic flows (volume, speed, and direction) change depending on the present posture to compensate the functional necessities of the body in real time, both for the head and the trunk and for the limbs [42-43]. These variations and liquid movements improve the biotensegretive and myofascial functions, protect the organs, and allow a better immune defence. There is a direct relation between the strength expressed by muscles and the quantity/speed of arterial blood used [44]. An artificially-induced reduction of the quantity of blood that reaches the musculature (e.g., during sports training) makes protein synthesis easier, with increasing muscle hypertrophy [45]. The venous return is facilitated when the limbs are positioned higher than the heart, so that the duty of the right atrium to receive venous blood is made easier [46]. Venous fluctuations with respect to postural changes may put some outflows in trouble. In the supine position, internal jugular veins are open and allow outflows from the cerebral system (also facilitated by breathing) [47]. In orthostatism, internal jugular veins collapse, and the venous outflow is redirected towards vertebral veins and up until it reaches the superior vena cava [47]. The lymphatic movement towards a specific district or tissue can not only mean a postural change, but also an immunological necessity. Lymphatic structures react to the inflammatory stimulus by increasing the drainage and the quantity of lymph, probably to make up for liquid loss from blood vessels. Unfortunately, if the cause that produces inflammation isn’t solved, the increase of lymph becomes counterproductive (secondary lymphatic edema) [48]. The new theoretic model, Rapid Adaptability of Internal Network (RAIN), tries to complete the biotensegretive model of the solid fascia, and suggests that it achieves its maximal functionality with the liquid fascia. The liquid fascia has a high variability in the pressures at which it flows, both at rest and during postural changes, in order to improve the continuum of the solid fascia, concerning the movement, function, and the shape. RAIN reminds us of the liquid tissue and, as an acronym, it highlights the rapid adaptability of blood and lymph inside of the net of the solid fascia. The liquid fascia is another element of discontinuous compression inside the biotensegretive myofascial model. Compared to the biotensegrity model, the new model is a closed system, thanks to the epidermis, which is part of the solid fascia [1]. In the first there is no skin and is based on an architectural model; since there is no skin, it is an open model. This is the first article in the world that considers the liquid fascia inside of the better-known biotensegretive model of the solid fascia. This text can be of inspiration for other researchers, and can help improve manual and therapeutic approaches; it can also aid our comprehension of bodily dysfunctions, thanks to a different perspective counter to the general view of the symptomatic framework.

## Conclusions

The article reviews definitions of the solid fascia and the liquid fascia, and tries to integrate the concept of biotensegrity into a new model. The final aim is to improve the vision of the body system including the blood and lymph, because in the previous model these two liquid elements are not taken into consideration. The biotensegretive model describes the principle of self-regulation in the living, where constant tension is represented by musculature, and bones/joints play the role of discontinuous compression. The name given to the new model is Rapid Adaptability of Internal Network (RAIN). We think that the liquid fascia is another element of discontinuous compression inside the biotensegretive myofascial model.
